# Diguanylate Cyclases in *Vibrio cholerae*: Essential Regulators of Lifestyle Switching

**DOI:** 10.3389/fcimb.2020.582947

**Published:** 2020-10-22

**Authors:** Sumit Biswas, Om Prakash Chouhan, Divya Bandekar

**Affiliations:** ViStA Lab, Department of Biological Sciences, Birla Institute of Technology and Sciences (BITS), Pilani–KK Birla Goa Campus, Goa, India

**Keywords:** biofilm, GGDEF, cyclic-di-GMP, virulence, diguanylate cyclase

## Abstract

Biofilm formation in *Vibrio cholerae* empowers the bacteria to lead a dual lifestyle and enhances its infectivity. While the formation and dispersal of the biofilm involves multiple components—both proteinaceous and non-proteinaceous, the key to the regulatory control lies with the ubiquitous secondary signaling molecule, cyclic-di-GMP (c-di-GMP). A number of different cellular components may interact with c-di-GMP, but the onus of synthesis of this molecule lies with a class of enzymes known as diguanylate cyclases (DGCs). DGC activity is generally associated with proteins possessing a GGDEF domain, ubiquitously present across all bacterial systems. *V. cholerae* is also endowed with multiple DGCs and information about some of them have been pouring in over the past decade. This review summarizes the DGCs confirmed till date in *V. cholerae*, and emphasizes the importance of DGCs and their product, c-di-GMP in the virulence and lifecycle of the bacteria.

## Introduction

### *Vibrio cholerae*: Dual Lifestyle and Biofilm

Formation of biofilm enables the bacteria to survive and propagate despite the presence of antibiotics or other external stress. *Vibrio cholerae* is no exception. This bacterium adopts two different lifestyles—the motile pathogenic form in the human host, and the sessile form in waterbodies existing in associated biofilms. The biofilm allows the bacteria to survive nutrient limitations, fluctuations in oxygen levels, and massive changes in osmolarity (Rodney, 2002; Tischler and Camilli, 2004; Waters et al., 2008). Additionally, it also allows changes in the bacterial proteome by inducing favorable genes or suppressing unfavorable genes in order to adapt better.

Biofilm formation in *V. cholerae* is a three-step cyclic process, involving (a) surface attachment, (b) colony formation, and (c) dispersal. In the initial step (surface attachment), motile *V. cholerae* scan solid surfaces—with a preference for the chitinous exoskeleton of zooplanktons or phytoplanktons (Tamplin et al., 1990; Rawlings et al., 2007). The bacterium, powered by the single polar flagellum (with a Na^+^-driven motor and regulated by the Flh proteins) seeks a suitable surface (Echazarreta and Klose, 2019), and has been suggested to be quite selective in assaying the surface before selecting it for attachment (Utada et al., 2014). The Mannose-Sensitive Haemagglutinin type 4 surface pili (MSHA-pili) contribute to strong surface attachment during the initial attachment steps (Watnick et al., 1999; Wong, 2016).

After multiplication and the progression of colony formation, the size of the average member cell keeps on decreasing to increase the compaction in the biofilm. The size decreased from 2.4 μm (Drescher et al., 2016) at the beginning of biofilm to 1.8 μm for cellular communities having ~1,000 cells (Wong, 2016). Consequently, interbacterial distances in the biofilm matrix also show a significant decrease. The directionality of colony growth also changes incrementally with increase in colony size—while the initial growth is only one dimensional, growth happens in all three directions when cell number crosses 200. It is during this three-dimensional growth phase, the extracellular matrix composed of polysaccharides, proteins, and a small amount of nucleic acids (Joachim and Karl, 2002; Wong, 2016) is secreted. Vibrio polysaccharides (VPS) are essential for keeping the cells together and maintenance of the 3D structure. Proteins of the extracellular matrix, *viz.*, RbmA, RbmC and Bap1 play critical roles in the biofilm as well. The RbmA protein has been implicated in cellular adhesion, architecture and biofilm stability process, while the RbmC secreted on the outer surface of the cells creates flexible scaffolds where the cells can grow and multiply. The Bap1 protein maintains pellicle strength and hydrophobicity allowing the biofilm to propagate at the water-air interface (Römling et al., 2013; Hay and Zhu, 2015).

The last phase of biofilm formation is the dispersal of the bacterial cells from the biofilm to search and colonize a new substratum when conditions are favorable. Environmental conditions such as high/low oxygen level, the concentration of phosphate, Ca^2+^, etc, have negative effects (inhibition of *vps* gene transcription) on biofilm formation and induce the dispersal of the *V. cholerae* biofilm (Colwell and Huq, 1994; Hay and Zhu, 2015). Atleast two deoxyribonucleases and the Xds protein have also been reported to play crucial roles in biofilm dispersal (Römling et al., 2013; Sisti et al., 2013). The degradation of biofilm and extracellular matrix is induced by various environmental signals and other proteins, many of which are yet to be elucidated.

## Regulation of Biofilm Formation in *V. cholerae* and Pathogenesis

Formation of the biofilm comes at a premium—the amount of resources diverted and spent toward the formation is substantial, but the benefits are huge. Being able to thrive in adverse conditions accords the bacterium a different strategy for survival. Therefore, the process needs to be highly regulated and that is how it happens, with the interplay of various factors. In *V. cholerae*, transcriptional activators, repressor proteins and sigma factors RpoS and RpoE have been demonstrably involved in the process (He et al., 2012).

The structural genes for VPS synthesis have been reported to be essential for exopolysaccharide biosynthesis and biofilm formation (Yildiz and Schoolnik, 1999). These genes, located on *vps-1* (*vpsA* to *vpsK*) and *vps-2* (*vpsL* to *vpsQ*) operons, are positively regulated by VpsR and VpsT, while HapR negatively regulates the expression of the *vps* genes, and the positive regulators VpsR and VpsT themselves (Casper-Lindley and Yildiz, 2004; Beyhan et al., 2007). Both VpsR and VpsT bind directly to the *vps* promoter regions and have recognition sites in *vps-1, vps-2* and *vps-L* operons which act as regulatory sequences in the expression of extracellular polysaccharide and matrix protein synthesis (Fong et al., 2010). A recent report relates the activation of the *vps* operons to the concentration of VpsR as well as c-di-GMP (Hsieh et al., 2020) directly affecting the σ70 RNAP. Additionally, VpsT can act as a regulatory protein with recognition sequences for RbmA, whereas RbmC and Bap1 promoters also contain recognition sites for VpsR (Boyd and O'Toole, 2012; Zhao-Xun, 2015).

Activation of HapR is an important precursor to the process of biofilm dispersion. The N-terminal HTH domain of HapR directly binds to the *vps-2* operon at *vpsL* and *vpsT* (Jonas et al., 2008; Sudarsan et al., 2008). The activation of HapR is controlled by small molecules involved in the quorum sensing pathway. During the biofilm phase, lower concentrations of the quorum sensing molecules AI-2 and CAI-1 activate the transcription of quorum sensing regulatory RNAs (sRNA, via phosphorylation of RpoN and LuxO), which prevent the synthesis of HapR. With the increase in concentrations of AI-2 and CAI-1, LuxO is dephosphorylated, and the sRNAs are repressed, leading to the expression of HapR, eventually resulting in the dispersal of the biofilm (Tchigvintsev et al., 2010). Other negative regulators include the cAMP and the cAMP-receptor protein complex (Liang et al., 2007).

**Figure 1:**
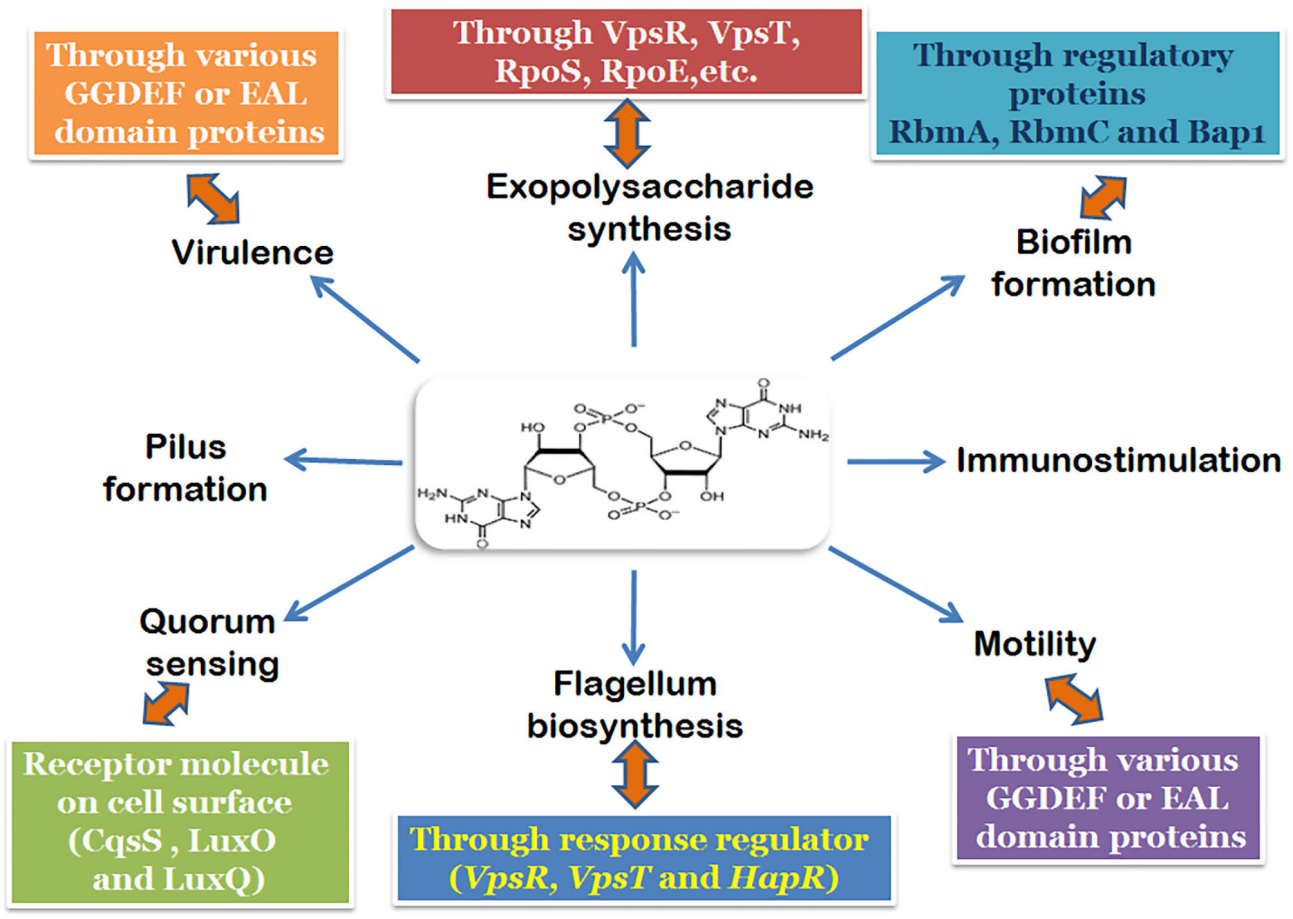
Regulatory pathways and components of the biofilm formation mechanism involving c-di-GMP in *V*. cholerae.

Intricately involved with all these regulatory elements, including those involved in pathogenesis is the secondary signaling messenger molecule cyclic-di-GMP (Figure 1; Watnick and Kolter, 2000; Tischler and Camilli, 2005). Both the biofilm activators, VpsT and VpsR can bind to c-di-GMP and has been shown to be responsive to fluctuations in the intracellular concentrations of c-di-GMP in *V. cholerae* (Krasteva et al., 2012; Hay and Zhu, 2015). An increase in the cellular c-di-GMP pool leads to the dimerization and activation of VpsT to induce biofilm formation (Shikuma et al., 2012). Similarly, allosteric activation of VpsR happens when the intracellular concentration of c-di-GMP rises. The activation of both VpsR and VpsT enhances the expression of genes essential for the formation of the biofilm. The third major component which responds to changes in c-di-GMP concentration is the σ^54^-dependent activator FlrA, which is linked to the expression of flagellar motility. Increased c-di-GMP levels lead to binding of c-di-GMP to FlrA, and inhibition of its activity which in turn diminishes flagellar gene expression (Srivastava et al., 2013). The dynamic extension and retraction of the MSHA pilus (Jones et al., 2015; Wang et al., 2016) is regulated by c-di-GMP via interaction with the ATPase MshE (Floyd et al., 2020). The role of c-di-GMP in the regulation of large adhesins which control reversible cell attachment during biofilm formation also highlights the essentiality of the molecule (Kitts et al., 2019). It is safe to state that c-di-GMP is a crucial and essential regulatory element for surface attachment and biofilm formation in *V. cholerae*.

Biofilm formation would therefore, be ideally associated with the loss of motility and switch toward the sessile, non-pathogenic lifestyle. However, the formation of biofilm is not just an essential ability which enhances the infectivity of *V. cholerae* (Zamorano-Sánchez et al., 2019), but also has been found to be crucial to the process of intestinal colonization. Interestingly, Xu et al. (2003) found that the expression of biofilm genes (*vpsA and rbmA)* was higher in rabbit ileal loop models. However, other biofilm-promoting genes like the *rbmC* and *bap1* did not seem to have any role to play in intestinal infection models (Fong et al., 2006), suggesting that the biofilms formed during intestinal colonization do not proceed beyond the RbmA-dependent primary cell aggregates (Silva and Benitez, 2016). Once in the intestine, the bacterium is exposed to multiple reverses like the effect of taurocholate salts in bile (Hay and Zhu, 2015) which is believed to degrade the VPS of the biofilm. Further repression of *vps* expression happens when the mucus layer is encountered, and the subsequent dispersal of the biofilm (Liu et al., 2007) results in the faster movement of the released bacterium in mucus. It has been postulated that the components of mucin might repress *vps* expression by actually regulating intracellular c-di-GMP concentrations (Liu et al., 2015) during *V. cholerae* infection. However, there has been no further elucidation of the interactions between mucin and c-di-GMP to explain the possible switch in the intestine.

## c-di-GMP and Diguanylate Cyclases in *V. cholerae*

Cellular c-di-GMP levels are regulated by the synthesis of activities of c-di-GMP by diguanylate cyclases (DGCs), and degraded by phosphodiesterases (Römling et al., 2013; Bandekar et al., 2017). Apart from *Mycobacterium smegmatis* (only two DGCs) (Kumar and Chatterjee, 2008), there is an abundance of DGCs in different bacterial systems (Römling et al., 2013; Chouhan et al., 2016). The multitude of functionalities regulated by DGCs and phosphodiesterases is very wide and even after years of investigation, the roles that they execute in these processes are not fully understood. The consensus is that the competitive action of the DGCs (and even the phosphodiesterases) results in the complex interactions between various pathways, but how or why these happen is yet to be elucidated. Even the response of DGCs to various extracellular signals and quorum sensing involves an intricate, network-modulated pathway, which might need years to unravel.

In *V*. *cholerae*, sensing environmental cues in the surrounding water or in the small intestine have been closely associated with fluctuations in the intracellular c-di-GMP pool. Generally, an increase in the levels of cellular c-di-GMP is associated with the suppression of the virulence genes in *V*. *cholerae* (Tischler and Camilli, 2005; Tamayo et al., 2007). Currently, it is accepted that the bacterium invades the gastrointestinal (GI) cavity with augmented levels of c-di-GMP, which are acted upon by the mucin components and eventually, the action of the phosphodiesterases bring down the c-di-GMP concentration (Koestler and Waters, 2014). During the late infection phase, though, there have been reports of a spurt in c-di-GMP concentration with expression of DGCs (Tamayo et al., 2007). The fluorescent visualization of the distribution of vibrios in the small intestine (Millet et al., 2014) has also brought to light the differential localizations in distinct niches along the small intestine, limited by the abundance of mucin. Together, these cues point to the following scenario—*V. cholerae* invades the GI tract with high levels of cellular c-di-GMP, which is brought down subsequently during the infective stage of the lifecycle. Once the bacteria is in the distal parts of the small intestine, where mucus is less abundant, c-di-GMP levels are raised again, as if in preparation for the life upon exit from the human host.

### Diguanylate Cyclases of *V. cholerae*

Diguanylate cyclases, responsible for c-di-GMP synthesis in bacteria, have been associated with a conserved GGD(/E)EF motif across different families (Ryjenkov et al., 2005). In line with the multiplicity of these proteins in bacteria, *V. cholerae* has been known to have 31 different proteins with a conserved GGD(/E)EF domain and 10 with a GGD(/E)EF and EAL (phosphodiesterase) domain in tandem distributed across its two chromosomes (https://www.ncbi.nlm.nih.gov/Complete_Genomes/c-di-GMP.html) (Conner et al., 2017). However, not all of these are associated with motility and/or biofilm formation, and some have not been demonstrated to have DGC activity. Generally DGCs have an active site (A site) where the synthesis of c-di-GMP takes place and a site for allosteric control (RXXD) which regulates the synthesis. We would elaborate on the few DGCs from *V. cholerae* which have been elucidated over the years.

#### CdgD

When the GGDEF domain was still named as a domain of unknown function (DUF), Yildiz et al. (2004) had identified five genes encoding proteins with GGDEF and GGDEF plus EAL domains which were differentially expressed between the smooth and rugose variants of *V. cholerae*. The proteins encoded by these genes were named Cdg A-E and assayed for their expression. Of these, the CdgD and CdgC deletion mutants showed significant alteration in the biofilm formation of the strains harboring them. While CdgD had a GGDEF domain along with a sensory PAS domain, CdE showed the presence of both GGDEF and EAL domains (Lim et al., 2006). While deletion of *cdgD* caused an increase in motility, *cdgC* mutants were associated with a 2.3-fold decrease in motility. CdgD was later characterized as a diguanylate cyclase and CdgC was responsible for negative regulation of VPS biosynthesis (Lim et al., 2007).

#### CdgH

Subsequently, (Beyhan et al., 2007) reported the activity of another protein with a predicted GGDEF domain, which they named CdgH. Overexpression of *cdgH* resulted in a high amount of c-di-GMP accumulation in the cell, which established CdgH as a diguanylate cyclase. Additionally, CdgH positively regulated the rugosity of the cell. The structure of CdgH is one of the two solved *V. cholerae* DGC structures, and displayed the presence of two N-terminal tandem periplasmic substrate-binding (PBPb) domains for signal recognition (Xu et al., 2017). Additionally, the same group had characterized several other predicted GGDEF domain proteins, which were not however DGCs.

#### VCA0965

A further DGC in *V. cholerae* was reported by the Waters lab in 2014 (Hunter et al., 2014). Interestingly, this protein did not have the conserved GGDEF motif, but had a degenerate AGDEF site. Significantly, expression of VCA0965 in *V. cholerae* was shown to cause a three-fold reduction in flagellar-based motility. This was noteworthy as many of the other predicted GGDEF proteins with conserved sequence did not show DGC activity, whereas VCA0965, despite its degenerate active site, could synthesize c-di-GMP.

#### VC0395_0300

**Figure 2:**
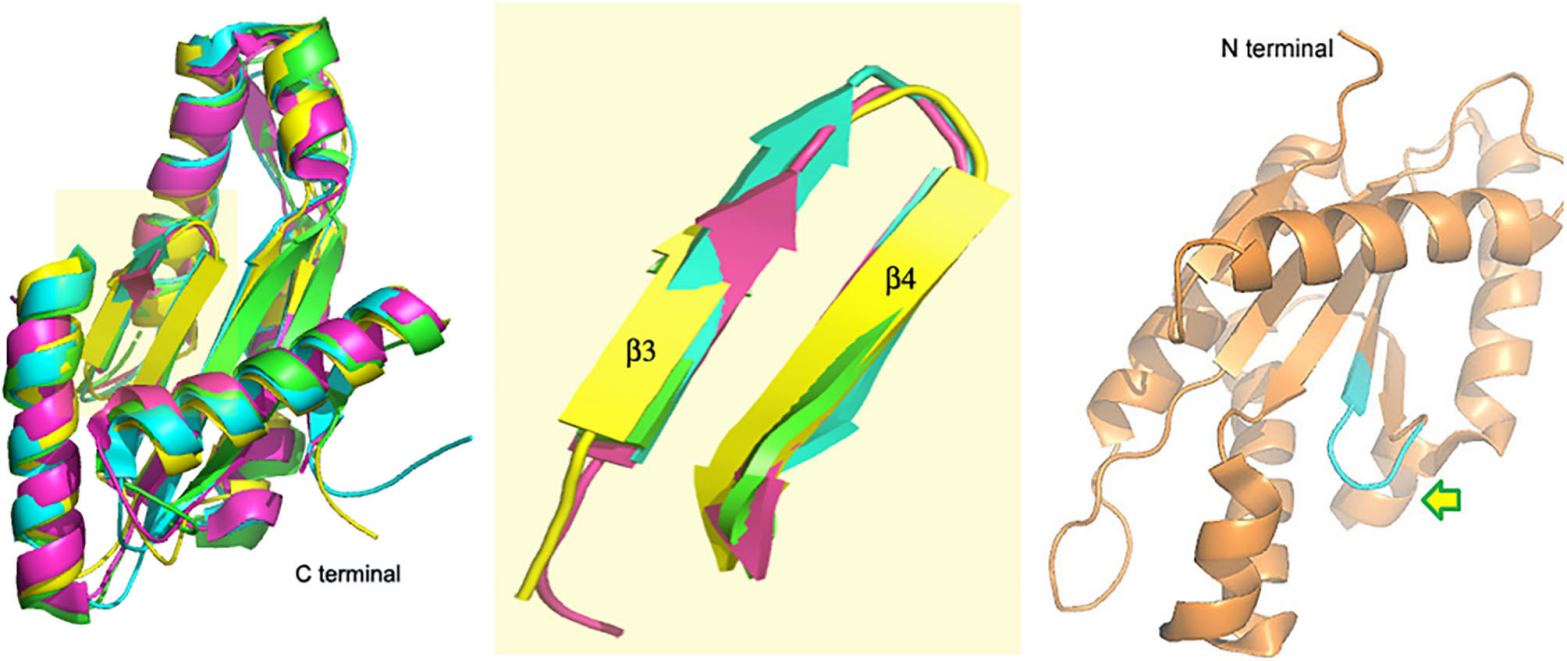
VC0395_0300_(161−321)_ (yellow, 6EIB) aligned with SadC (blue, 4WXW, *Pseudomonas aeruginosa*) and DGCs from *Thermotoga maritima* (green, 4URG) and *Bdellovibrio bacteriovorus* (pink, 6HBZ) Highlight: Alignment of GGDEF domains. Right: Active site of VC0395_0300 in cyan. Arrow shows direction of entry of GTP.

A DGC with a GGEEF domain was reported by our group in 2017 (Bandekar et al., 2017; Chouhan and Biswas, 2018). While VC0395_0300 was shown to synthesize c-di-GMP actively and had an essential role to play in the biofilm formation of *V. cholerae*, mutations at the central positions of the GGEEF sequence were detrimental to the functional activity of the protein (Chouhan et al., 2016). The structure of the protein though showed similar architecture (Figure 2) associated with diguanylate cyclases from other bacterial systems (Chouhan et al., 2020). Another deviation in this DGC was that it lacked the site for allosteric inhibition found in the other DGCs of *V. cholerae*, suggesting a different mode of inhibitory control in this DGC.

## Concluding Remarks

The secondary messenger c-di-GMP plays the most crucial role in the regulation of biofilm formation and motility of *V. cholerae*. The levels of intracellular c-di-GMP are modulated by a host of factors including the diguanylate cyclases from which these are synthesized, the phosphodiesterases which lead to their degradation, and other receptor molecules including several virulence genes. The abundance of GGDEF domains in bacterial species, coupled with the uncertainty around their function as diguanylate cyclases renders further complexity to the mechanism of action of this class of enzymes. To add to the conundrum, the ability of degenerate GGDEF domains to synthesize c-di-GMP and the variance of allosteric inhibitory mechanisms in the DGCs are also systems of interest. It has been hypothesized that the multiple DGCs don't fire in unison—one or a few of them might be expressed at a time, possibly in response to an environmental cue. The association of the DGCs with an extra sensory domain in most cases points to the interaction of the DGC with the extracellular environment. Elucidation of the modes of action of the other DGCs and their regulation vis-à-vis the sensory domain will lead to solving the enigma of multiplicity of the DGCs.

The hitherto unexplored role of c-di-GMP against the host immune system is also an area of intrigue which has been poorly explored. In mammals, c-di-GMP was found to activate the innate immune system by binding to STING (stimulator of interferon genes) (Burdette et al., 2011). However, how the host immune response affects the levels of intercellular c-di-GMP also needs to be explored and should open up newer areas of understanding of this signaling messenger. The observation of hyperinfectivity (a short-lived but elevated infectious state where the virulence gene expression is high) in biofilm-grown cells of *V. cholerae* in comparison to planktonic cells (Gallego-Hernandez et al., 2020), makes it extremely important to understand the mechanism of biofilm-formation in the bacteria.

## Author Contributions

SB, OC, and DB contributed to the drafting and writing of the manuscript. All authors contributed to the article and approved the submitted version.

## Conflict of Interest

The authors declare that the research was conducted in the absence of any commercial or financial relationships that could be construed as a potential conflict of interest.
